# Data on the structure, chemical state of silicon carbide synthesized by adiabatic cyclic compression in a chemical reactor

**DOI:** 10.1016/j.dib.2019.104868

**Published:** 2019-11-22

**Authors:** Boris Ezdin, Dmitriy Yatsenko, Valerii Kalyada, Alexandr Zarvin, Arkady Ichshenko, Aleksei Nikiforov, Pavel Snytnikov

**Affiliations:** a Novosibirsk State University, st. Pirogova 2, 630090 Novosibirsk, Russia; b Boreskov Institute of Catalysis, Siberian Branch of the Russian Academy of Sciences, Pr. Akad. Lavrentieva 5, 630090 Novosibirsk, Russia; c Limited Liability Company “NICOM”, st. Tereshkova 33, 630090 Novosibirsk, Russia

**Keywords:** Silicon carbide, Silicon, Synthesis from gas phase, Chemical compression reactor, Semiconductive nanomaterials, Cyclic flowing pyrolysis method, Nanoscale crystallites

## Abstract

The data presented in this article relate to the scientific article “Pyrolysis of a mixture of monosilane and alkanes in a compression reactor to produce nanodispersed silicon carbide” [1]. In the above article, a method is proposed for producing nanosized silicon carbide powder by compression in a cyclic process of a chemical reactor. Pyrolysis is initiated in mixtures of monosilane and hydrocarbon with argon. The article also presents the analysis data of the obtained product by X-ray diffraction and electron microscopy.

**Table:** Specifications Table

Subject	Chemical engineering, materials science, nanotechnology
Specific subject area	Silicon carbide nanopowders, silicon, chemical compression reactor
Type of data	Image Chart Graph Figure
How data were acquired	Hardware: • High-resolution transmission electron microscopy (HRTEM) JEM-2010 (JEOL). • ARL X'TRA powder diffractometer (Thermo Fisher Scientific) • Universal Gas Analyzer UGA-200 Databases: • Powder Diffraction File 4+ (PDF) • Inorganic Crystal Structure Database 2018 (ICSD) Software: • Topas (Bruker) • Gatan Digital Micrograph
Data format	Raw Analyzed
Parameters for data collection	Data were obtained on the synthesis of silicon carbide in a cyclic chemical reactor at maximum pressures up to 12 MPa. Used argon mixtures with precursors. The concentration of precursors did not exceed 10% of the total volume of the mixture. The temperature of the mixture at the inlet of the chemical reactor was 300 K
Description of data collection	The pressure and composition of the gaseous products were measured on-line by UGA-200 during the operation of the chemical compression reactor. HRTEM data are obtained at an accelerating voltage of 200 keV
Data source location	Department of Applied Physics, Faculty of Physics, Novosibirsk State University, Boreskov Institute of Catalysis SB RAS, Novosibirsk, Russia
Data accessibility	With the article
Related research article	B.S. Ezdin, D.A. Yatsenko, V.V. Kalyada, A.V. Ichshenko, A.E. Zarvin, A.A. Nikiforov, P.V. Snytnikov Pyrolysis of a mixture of monosilane and alkanes in a compression reactor to produce nanodispersed silicon carbide Chemical Engineering Journal https://doi.org/10.1016/j.cej.2019.122642

**Table:** 

**Value of the Data** • The obtained data expand the experimental facts base on silicon carbide nanopowders obtained from the gas phase using a chemical compression reactor. • The data can be used for qualitative and quantitative assessment of the structure, chemical state of silicon carbide powder obtained using adiabatic compression, as well as for comparison with the properties of nanopowders obtained by other methods. • The presented data on the method of production, structure and composition of nanopowders can be used to develop methods for modifying existing and obtaining new functional materials. • Data on the pressure, temperature and degree of processing of the feedstock are necessary for the further development of the adiabatic compression method to obtain nanopowders of other substances, and to evaluate the effectiveness of such processes.

## 1 Data

The data set of this article contains information on the characteristics of silicon carbide nanopowders, obtained by the method of adiabatic compression in a chemical reactor [2]. Data on parameters inside the compression chamber, pressure and temperature are given. In papers [[3], [4], [5]], the importance of knowing the law of variation of these quantities during the compression-rarefaction cycle is indicated.

**Fig. 1:**
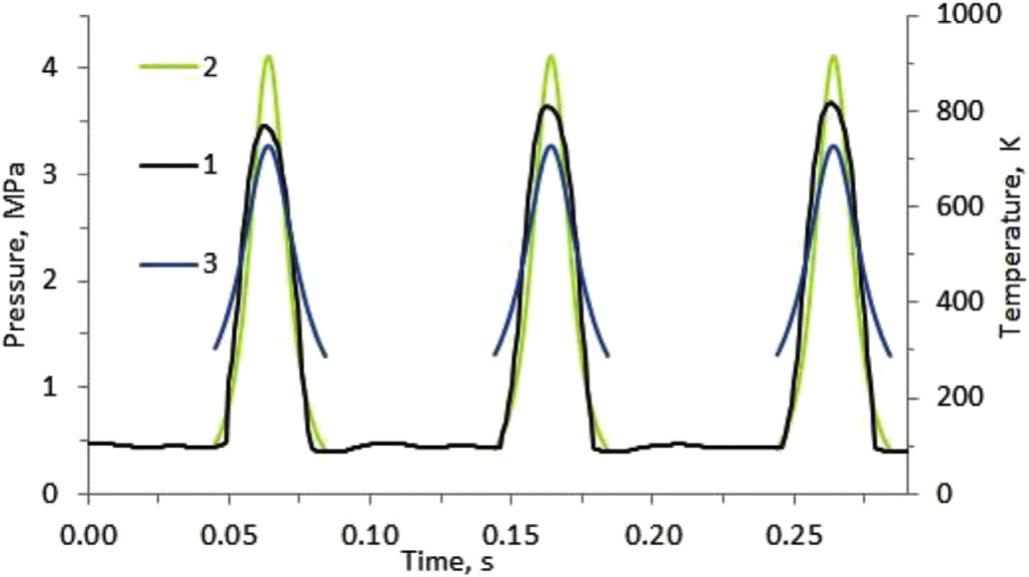
The change in thermodynamic parameters inside the compression chamber during three cycles of compression - rarefaction: experimental data on pressure (1). Calculated data in the adiabatic compression approximation of an ideal gas: pressure (2) and temperature (3).

The experimental data on the change in pressure inside the chamber during a period of three cycles of compression - rarefaction shows in Fig. 1. The black line is the experimental value obtained. The green line is the calculated dependence obtained in the approximation of the adiabatic ideal gas process, without taking into account the pressure drop during the opening of the exhaust valve. The experiment was performed using a crank mechanism. The calculation of the periodicity of cycles was obtained in the approximation of the harmonic law of motion of a piston with a frequency of 10 Hz. The temperature in the graph in Fig. 1 is marked with a blue line. The calculation was performed in the adiabatic compression approximation of a monatomic ideal gas according to T·V^γ^ = const and p·V^γ^ = const for initial data p = 0.45 MPa, T = 300 K.

Experiments on the synthesis of silicon carbide nanopowder were carried out at different maximum pressures. The pressure was varied by adjusting the opening of the exhaust valve.

**Fig. 2:**
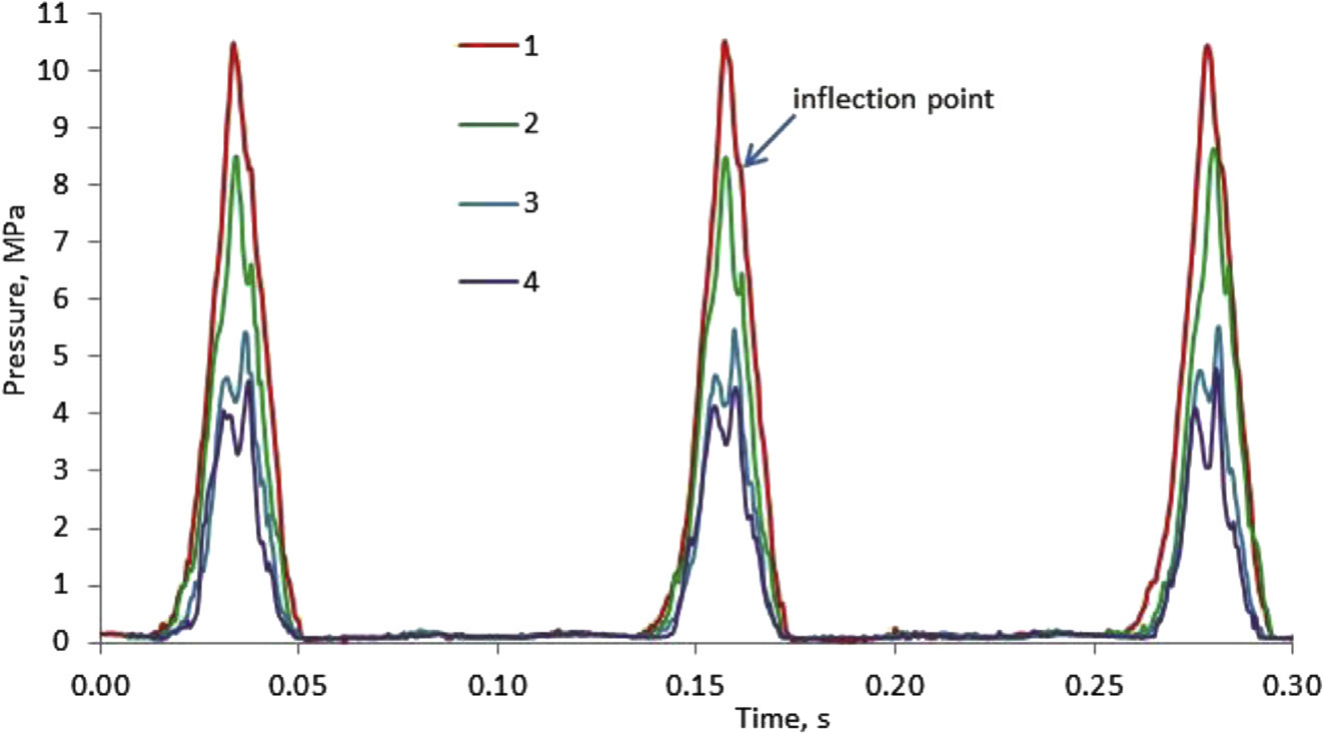
Pressure plots inside the compression chamber obtained for different opening modes of the exhaust valve. Maximum pressure: (1) - 10.5 MPa; (2) - 8.5 MPa; (3) - 5.5 MPa; (4) - 4.5 MPa.

Fig. 2 shows graphs of pressure changes caused by a change in the volume of the chamber due to piston movement from a point of maximum volume (commonly referred to in the theory of internal combustion engines as a bottom dead center (BDC)), to a point of minimum volume (top dead center (TDC)). The appearance of a characteristic additional pressure peak (at pressures of 4.5 and 5.5 MPa) or the inflection point of the graph at higher maximum pressures (10.5 MPa) in the compression-rarefaction cycle is due to exothermic chemical reactions in the mixture. When compressing inert gases, the pressure graph is symmetrical. Changing the maximum pressure in the chamber from 4.5 MPa to 10.5 MPa made it possible to determine the optimal operating conditions of the reactor to obtain maximum performance in the compression-rarefaction cycle. For pressures of 4.5 MPa and below, the resulting product could consist of two phases - silicon carbide and silicon. The experiments showed an increase in the selectivity of the synthesis reaction of silicon carbide up to two times. The influence of other initial data (composition, temperature, compression rate, etc.) on the synthesis processes is the subject of another scientific article.

The passing of the synthesis reaction of SiC was controlled on-line using a universal gas analyzer UGA-200, connected by a capillary tube to the tank collecting products. The appearance of a larger amount of hydrogen indicated the passage of the synthesis reaction, and the disappearance or a small amount of the initial reagents (monosilane and hydrocarbons) indicated the completeness of the processing reaction.

**Fig. 3:**
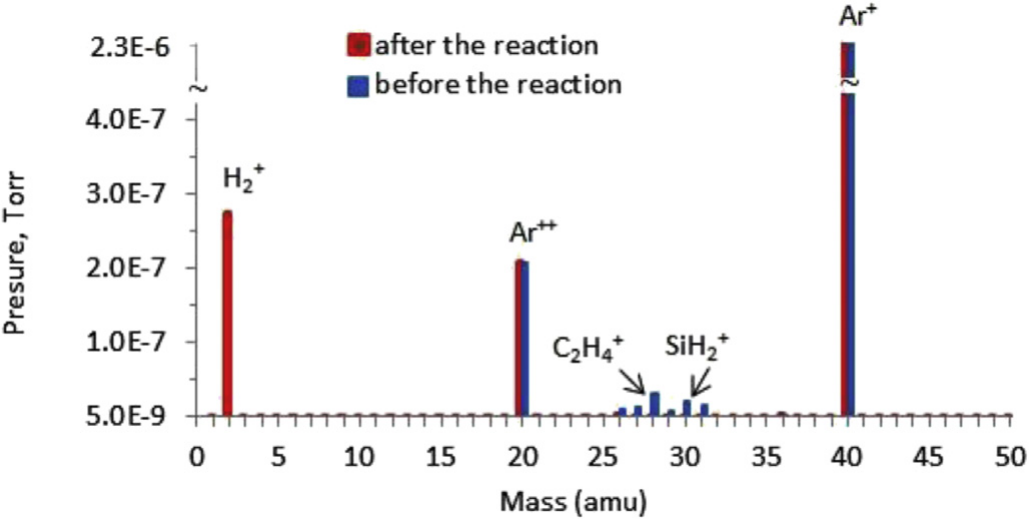
The data of the universal gas analyzer UGA-200: processing of a gas mixture of H2 - 10%, Ar - 90%.

An example of a graph of the qualitative composition of gaseous reaction products (red color is used) is shown in Fig. 3. The composition of the initial gas mixture: 2.5% SiH_4_ + 2.5% C_2_H_4_ + 95% Ar (blue color is used). Processing of the initial SiH_4_, C_2_H_4_ precursors is almost complete.

**Fig. 4:**
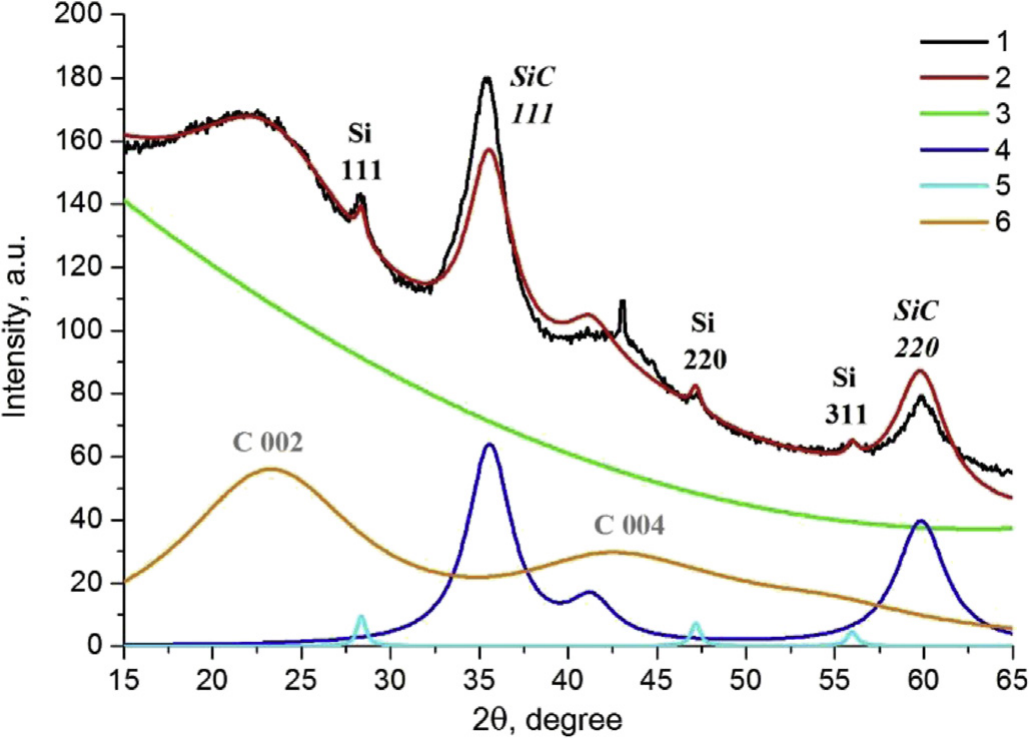
Experimental diffraction pattern (1) for the sample obtained at pressures of 7.8 MPa and Rietveld simulation (2): background (3), silicon carbide (4), silicon (5), amorphous graphite (6).

The diffraction data showed the presence of crystalline silicon and silicon carbide phases. Carbon is probably in the amorphous graphite phase and corresponds to a halo in the region of 20–25° 2θ (Fig. 4).

In the article [1] to determine the phase content, crystallite size and unit cell parameters, the diffraction patterns were simulated using Pauli method [7] by the Topas program. The initial structural data were taken from ICSD: for silicon card is 52266, silicon carbide - 24217 and carbon - 53781.

**Fig. 5:**
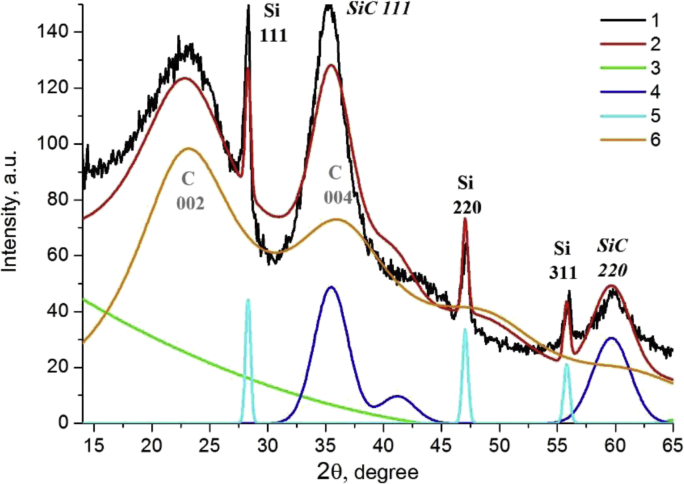
Experimental diffraction pattern (1) for the sample obtained at pressures of 4.6 MPa and Rietveld simulation (2): background (3), silicon carbide (4), silicon (5), amorphous graphite (6).

The Pauli method was used in the article [1] because an attempt to apply the Rietveld method [8] did not lead to good profile fitting: a comparison of theoretical and experimental diffraction patterns for samples of a mixture of 2.5% SiH_4_ + 2.5% C_2_H_4_ + 95% Ar obtained at a pressure of 7.8 MPa and at 4.6 MPa by the Rietveld method are shown in Fig. 4, Fig. 5, respectively.

**Fig. 6:**
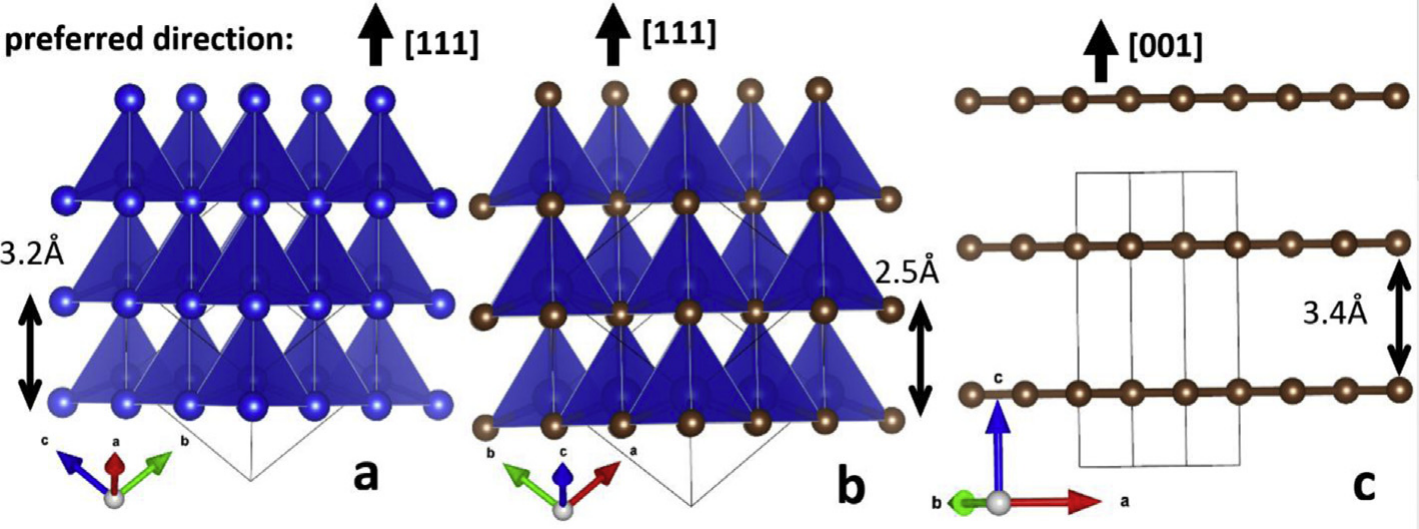
Images of structures for silicon - (a), silicon carbide - (b) and graphite - (c).

It is seen that the model describes the experiment unsatisfactorily (peak intensities do not correspond to theoretical calculations). This is probably due to the fact that at nanometer sizes the anisotropic structure of crystallites affects their habitus and the morphology of nanocrystallites does not correspond to large-crystalline state. Thus, the structure of silicon and silicon carbide represent the close cubic packing which has the preferred direction [111], Fig. 6a, b. The graphite structure is layered, the graphene grids are arranged along the [001] direction of Fig. 6c.

**Fig. 7:**
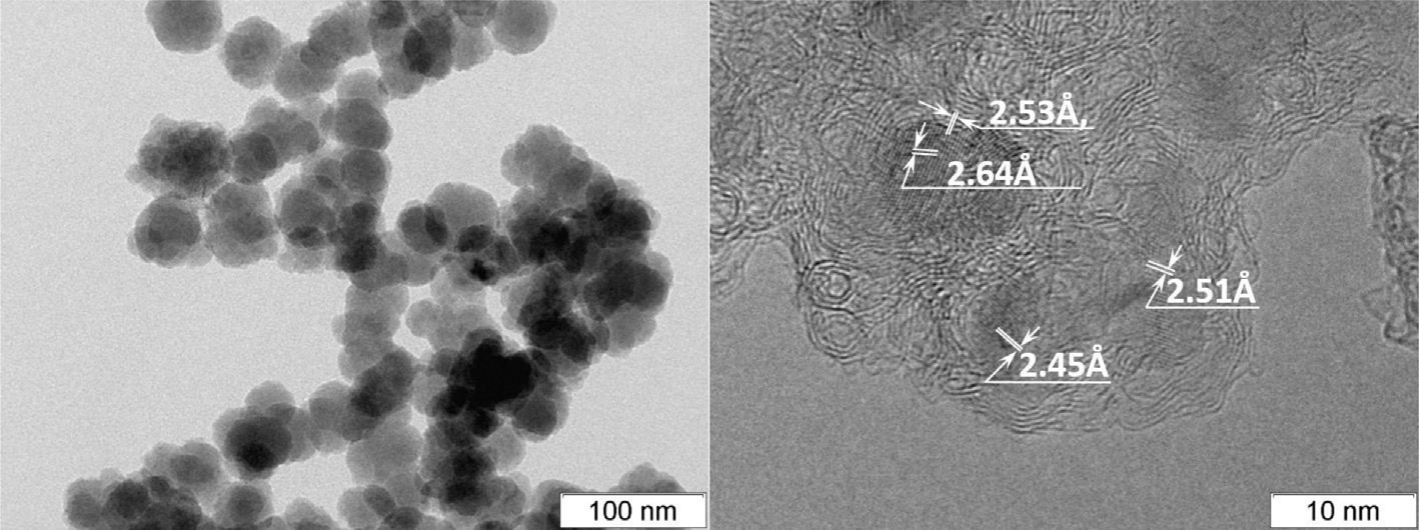
Electron microscopic images of particles of a sample of nano-dispersed silicon carbide.

**Fig. 8:**
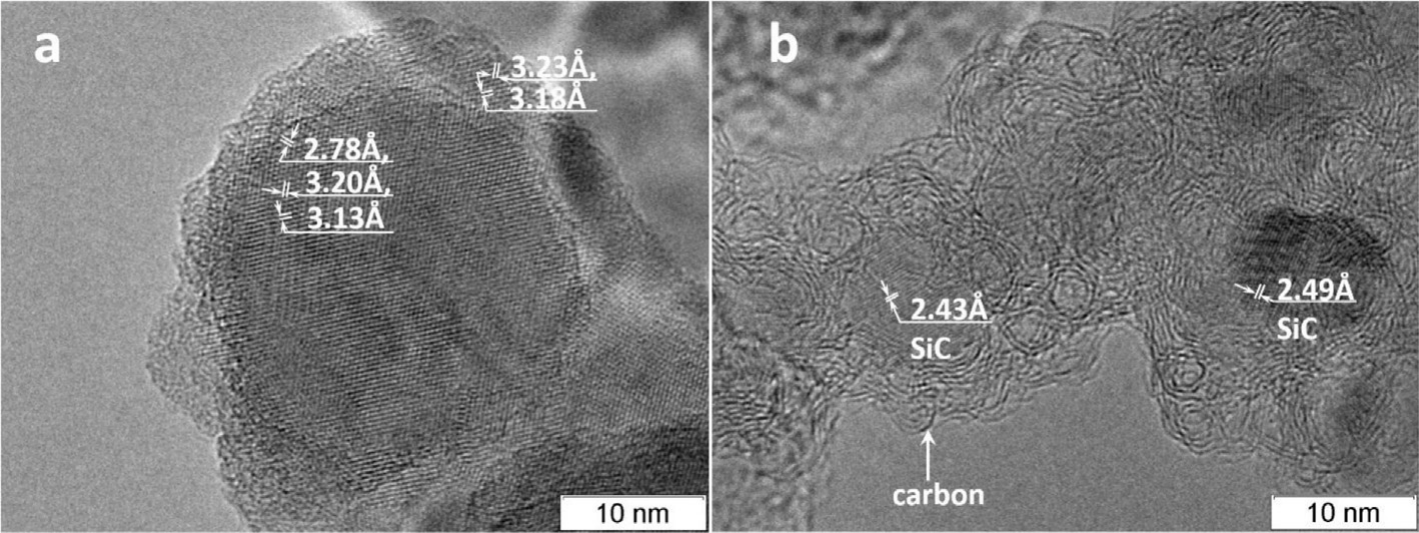
Nanoscale crystallites Si - (a) and Core-shell particles - SiC 3–10 nm in size coated with carbon 2–5 nm thick - (b).

The interplanar distance along [111] for silicon corresponds to 3.2 Å (Fig. 6a), for silicon carbide 2.5 Å (Fig. 6b) and for carbon interplanar distance along the [001] direction is 3.4 Å (Fig. 6c). According to these interplanar distances characteristic of structures, it was possible to estimate the morphology and identify the charge arrangement of the phases according to electron microscopic images (Fig. 7, Fig. 8).

The sample is represented by two morphologically different types of particles: rounded well-crystallized particles of silicon carbide (more contrast in the image) and less contrasting nano-sized particles forming extended dendritic aggregates. The composition of the initial gas mixture is close to the stoichiometric balance.

Fig. 8a shows crystalline silicon particles of size 20–30 nm with an amorphized surface layer obtained from a carbon-poor reaction mixture. Fig. 8b shows a photograph of the Core-shell structure. The core is formed by SiC crystallites 3–7 nm in size, covered with several layers of graphene. A mixture of monosilane and argon with acetylene is used.

## 2 Experimental design, materials, and methods

Silicon carbide nanopowder was synthesized in a cyclic chemical reactor by compressing a mixture of monosilane and some hydrocarbons with argon. The working pair of piston-cylinder is made of aluminum alloy and is protected by a ceramic coating obtained by the method of microarc oxidation [6].

The study of the obtained samples was carried out by HRTEM on a JEM-2010 electron microscope (JEOL, Japan) with an accelerating voltage of 200 kV and a resolution of 0.14 nm. Digital processing of the obtained electron microscopic images with the calculation of the observed interplanar distances according to the Fourier analysis of the area was performed using the Gatan Digital Micrograph program.

Diffraction patterns were obtained on a X'TRA powder diffractometer (Thermo, Switzerland, vertical θ/θ geometry, a Bragg-Brentano focusing, point semiconductor detector). The radiation source is an X-ray tube with a copper anode, the average radiation wavelength CuKα = 0.15418 nm. Generator current 45 mA, voltage 35 kV. The survey range is 15–65° at 2θ, the pitch is 0.075°, the accumulation time at the point is 5 seconds.

Nanoscale materials have a low density and, accordingly, miss a significant amount of x-ray radiation. When conducting X-ray studies on the reflection, the incident rays pass through the sample and are scattered on the cell. This leads to the fact that diffraction from it makes a significant contribution to the diffraction pattern and distorts the obtained data. Thus, standard plastic cuvettes supplied with serial diffractometers are unsuitable for determining the amorphous component of biopolymers. Therefore, special non scattering single-crystal cells were used in the work.

A mixtures of monosilane (2.5–5%), hydrocarbon (2.5–5%) with argon (90–95%) was used. Acetylene, ethylene or propane was used as a hydrocarbon. The hydrocarbon was used in an amount determined by the stoichiometric balance for the synthesis of silicon carbide. Also investigated options for excess and lack of hydrocarbons. The reagents were fed at a temperature close to 20 °C. The repetition rate of compression - rarefaction cycles was about 10 Hz.
